# Functional Characterization of Individual- and Mixed-Burgundian *Saccharomyces cerevisiae* Isolates for Fermentation of Pinot Noir

**DOI:** 10.3390/molecules20035112

**Published:** 2015-03-19

**Authors:** Emily Terrell, Margaret A. Cliff, Hennie J. J. Van Vuuren

**Affiliations:** 1 Wine Research Centre, Faculty of Land and Food Systems, University of British Columbia, Vancouver, BC V6T 1Z4, Canada; E-Mail: eterrell3@gmail.com; 2 Pacific Agri-Food Research Centre, Agriculture and Agri-Food Canada, Summerland, BC V0H 1Z0, Canada

**Keywords:** wine, *S. cerevisiae*, fermentation, volatile compounds, novel yeast strains

## Abstract

Pinot noir has traditionally been fermented by native flora of multiple yeasts producing a complex combination of aromas and flavors. With the use of industrial dry yeasts, winemakers gained enological reliability and consistency in their wines, but lost diversity and complexity. This research evaluated the use of co-culturing yeasts to fulfill this dual role. Fermentations of Burgundian *Saccharomyces cerevisiae* isolates and their mixtures were evaluated for their enological characteristics and production of volatile compounds, at 22 °C and 27 °C. The novel isolates were genetically unique and enologically equivalent to the industrial strains. Analysis of variance and principal component analysis of 25 headspace volatiles revealed differences among the yeasts and between the fermentation temperatures. Wines from the mixed-Burgundian isolates were most similar to one another and could be differentiated from the industrial strains at both 22 °C and 27 °C. Mixed-Burgundian wines at both temperatures had higher concentrations of ethyl esters and acetate esters, compared to the industrial strains which had higher concentrations of higher alcohols at 27 °C and higher concentration of other ethyl esters at 22 °C. Given the unique profiles of the co-cultured wines, this research offers winemakers a strategy for producing wines with unique and more complex characters without the risk of spontaneous fermentations.

## 1. Introduction

Pinot noir is well known as one of the most complex and revered red wine grape varietals. As Pinot noir’s popularity grows, there is an increasing demand for fermentation products designed to promote varietal-specific aromas and flavors as well as the complexity that some wine aficionados believe has deteriorated with the widespread use of single commercial yeast starter cultures. Recent advances in wine biotechnology may provide the best of both worlds: yeast products that perform enologically yet produce premium Pinot noirs redolent of the finest Old World techniques.

This search for premium yeast products has gone in several directions: (i) some scientists have utilized modern biotechnology and developed genetically modified yeast strains [1], (ii) others have captured the beneficial aspects of traditional spontaneous fermentation using mixed strain fermentations [2,3,4,5] and (iii) yet others have worked with native yeasts, not necessarily in spontaneous or mixed cultures to improve the aroma/flavor of fermented foods [6]. In such cases, novel yeast products must not only possess unique genetic traits or provide exceptional complexity, but must also meet the wine yeast phenotypic expectations that have evolved over the past 50 years of wine research. These enological traits have been grouped into two classes in the literature; technological traits that influence the efficiency of the fermentation process and qualitative traits that affect the chemical composition and the sensory profile of the finished wine [7].

The fermentation properties of wine yeast strains represent one subset of technological traits. Desirable fermentation properties include rapid initiation of fermentation, low nitrogen requirements, high fermentation efficiency, high osmotic stress tolerance, growth at high and low temperatures, moderate biomass formation and high ethanol tolerance [8]. Additional technological traits beyond fermentation properties are also desirable in wine yeast strains, including genetic stability, capacity for genetic marking, killer phenotype, low foam production, flocculation, high sulfur dioxide tolerance, low sulfur dioxide binding, compact sediment formation and resistance to desiccation and proteolytic activity [8].

Qualitative wine yeast traits directly affect the aroma or flavor of the finished wine. This encompasses the synthesis and liberation of a number of compounds, including acetaldehyde, acetic acid, sulfur compounds, higher alcohols and esters [8,9]. These compounds are currently understood to varying degrees in terms of their synthetic pathways, desirable concentrations, sensory impact and influence on other volatiles.

Despite the popularity of Pinot noir, research has not been particularly successful in elucidating the key aromatic compounds responsible for its varietal profile. Moio and Etievant [10] identified ethyl anthranilate, ethyl cinnamate, ethyl 2,3-dihydrocinnamate and methyl anthranilate as important Pinot noir odourants, although subsequent quantification of these compounds in 33 Pinot noir wines by Aubry *et al.* [11] revealed very low average concentrations that did not exceed the known thresholds of ethyl cinnamate and methyl anthranilate in water [12]. Aside from these esters, ethyl and methyl vanillate, acetovanillone, 3-methylthio-1-propanol, 2-phenylethanol, benzyl alcohol and 3-methylbutanoic, hexanoic, octanoic, and decanoic acids have been identified as potentially important in Pinot noir wines [13]. However, the characteristic aroma of Pinot noir is due to the combination of compounds derived from the grape, produced from the microflora (yeast, bacteria) and synthesized from primary and secondary metabolites [14].

As the understanding of the yeast volatiles continue to advance, so will the development of premium fermentation products designed to capture the benefits of improved flavor or flavor complexity from yeast fermentation. One such approach has been the investigation and characterization of mixed strain and mixed species inocula.

While the contribution of non-*Saccharomyces* yeast species to wine production has been the topic of much research [15], only a few studies have focused on the effects of deliberately mixing *S. cerevisiae* strains during fermentation [16]. By mixing yeast strains known to differ in their liberation of specific thiols, King *et al.* [16] were able to demonstrate that the volatile thiol content and the sensory profiles of wines differed in mixed *S. cerevisiae* culture beyond the effects of each individual yeast strain, suggesting a synergistic effect. Another study by Howell *et al.* [17] confirmed that the unique volatile profiles created by mixing *S. cerevisiae* strains during wine fermentation cannot be replicated by fermenting each strain individually and then blending the resulting wines. These findings, along with the simultaneous isolation of a number of novel *S. cerevisiae* strains from a premium vineyard in Burgundy led to the hypothesis that fermenting Pinot noir grape must with mixed ratios of these Burgundian isolates will result in unique volatile profiles that may be unique and/or more complex than those associated with fermentation by industrial or individual-Burgundian yeasts of *S. cerevisiae*.

To this end, research was undertaken: (i) to document the genetic uniqueness of the three new Burgundian isolates (A1, A2, A3) using genetic fingerprinting and phenotype characterization, (ii) to demonstrate that these isolates were enologically equivalent to industrial yeast strains for winemaking and (iii) to compare the volatile profiles of Pinot noir wines fermented individually and in mixtures to industrial strains fermented at 22 °C and 27 °C.

## 2. Results and Discussion

### 2.1. Genetic and Phenotypic Characterization of Wine Yeast Strains

**Figure 1:**
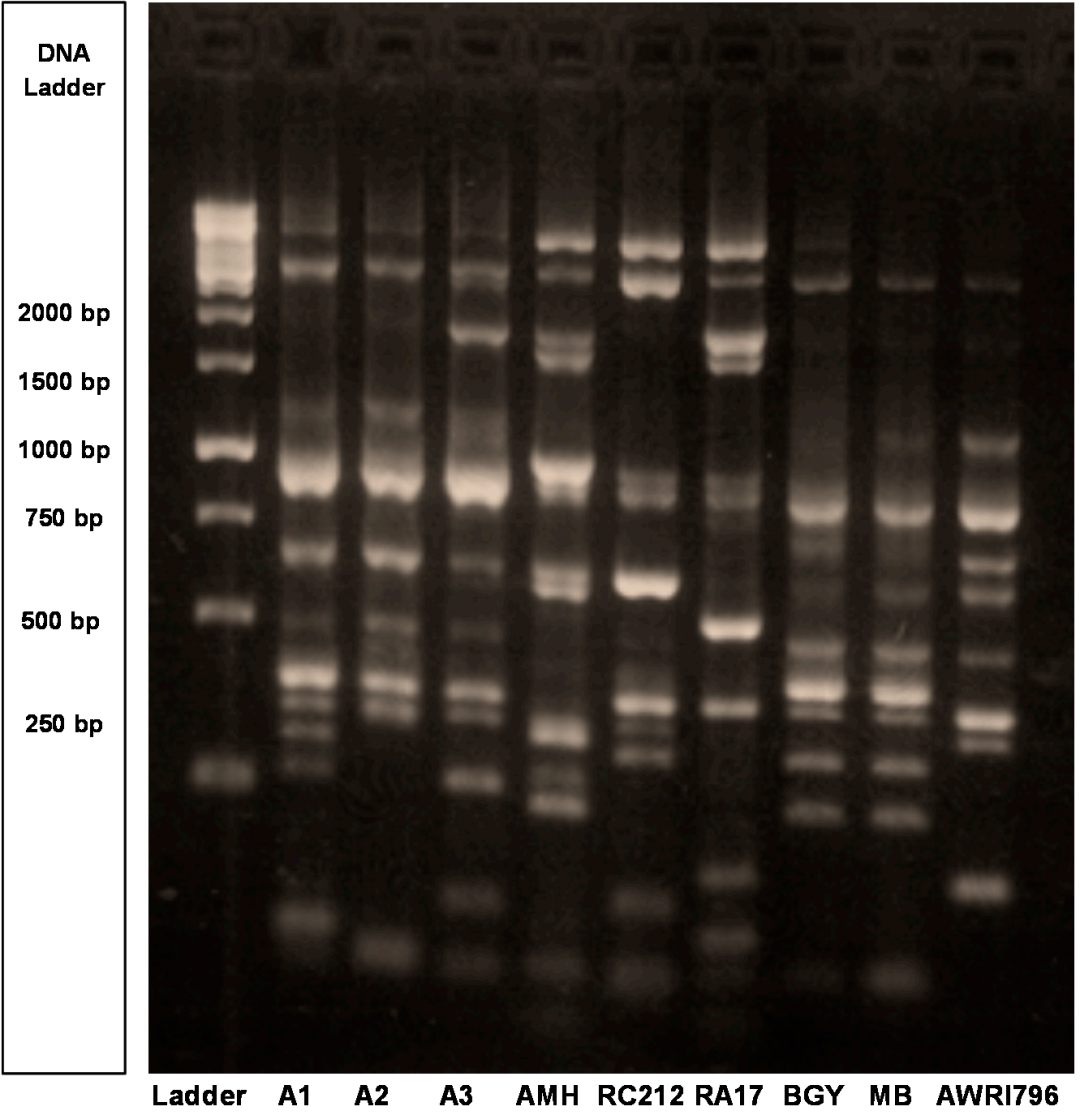
Genetic fingerprints of three Burgundian *S. cerevisiae* isolates (A1, A2, A3) and six industrial *S. cerevisiae* yeast strains [Enoferm Assmanshausen (AMH), Enoferm Burgundy (BGY), Lalvin RA17 (RA17), Lalvin Bourgorouge (RC212), Maurivin B (MB), Australian Wine Research Institute 796 (AWRI796)]. The δ sequence typing of all strains is shown relative to the 1 kilobase (kb) DNA ladder obtained from Fermentas (Thermo Fisher, Burlington, ON, Canada). Since the genetic fingerprint and fermentation data for BGY and MB were identical, MB was excluded from further analysis.

Genetic and phenotypic characterization revealed the genetic uniqueness of the Burgundian isolates (A1, A2, A3) and their compatibility with one another in mixed culture fermentations. PCR-based genetic fingerprinting [18] differentiated the three Bugundian isolates from six industrial (commercial) strains (Figure 1). All strains had unique banding patterns except Enoferm Burgundy (BGY) and Maurivin B (MB) which were identical. Therefore, MB was excluded from further analysis (Figure 1). As such, they were genetically different from one another and would be expected to have different enological characteristics [19].

**Figure 2:**
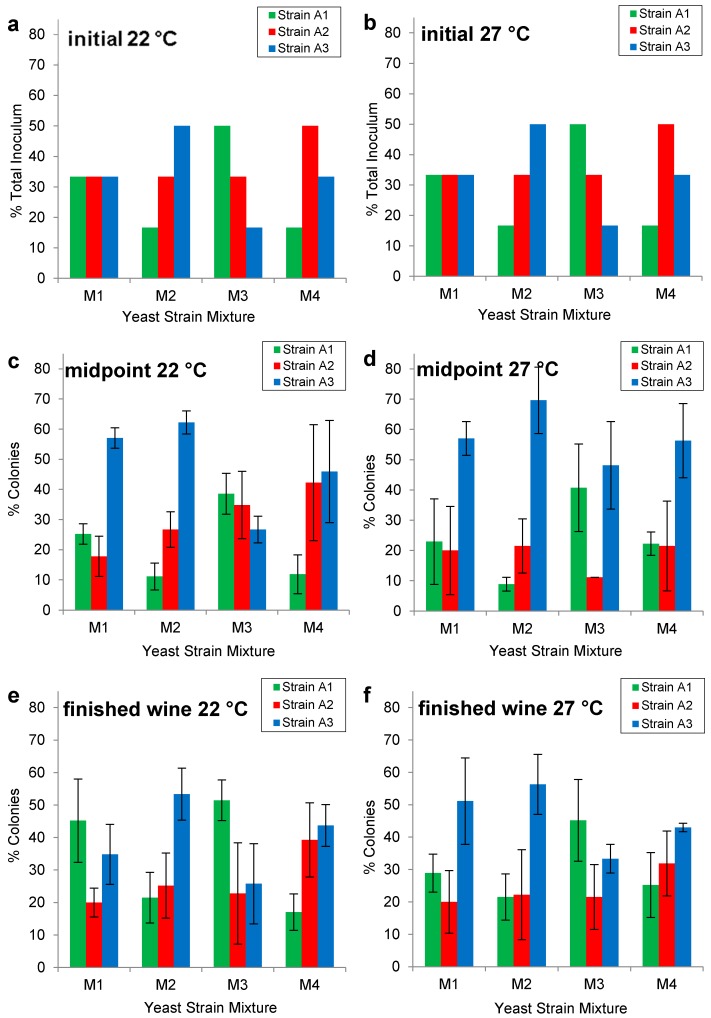
Initial inoculation ratios of Pinot noir fermentations co-cultured with Burgundian isolates (A1, A2, A3) in mixtures M1 (1:1:1), M2 (1:2:3), M3 (3:2:1), and M4 (1:3:2) at (**a**) 22 °C and (**b**) at 27 °C. Mixed strain populations at the midpoint (~6.0% v/v ethanol) (**c**) at 22 °C and (**d**) 27 °C and end of fermentation (~13.5% v/v ethanol) (**e**) at 22 °C and (**f**) at 27 °C. Yeast populations were quantified using colony PCR in conjunction with δ sequence typing on 45 colonies for each of three biological replicates.

The Burgundian isolates were identified as killer positive (K+) phenotype. As such they would be expected to kill wild yeast strains, predominate in individual strain fermentations and be able to be co-cultured with one another [20,21]. The initial strain ratios for each of the mixed cultures (M1, M2, M3, M4) at 22 °C and 27 °C are shown in Figure 2a,b. Colony PCR in conjunction with δ sequence typing revealed the strain ratios at the midpoint (22 °C Figure 2c; 27 °C Figure 2d) and end (22 °C Figure 2e; 27 °C Figure 2f) of fermentation. The mixed culture M2 approximately maintained the inoculated yeast ratios at the midpoint (Figure 2c,d) and immediately following fermentation (Figure 2d,e) at 22 °C and 27 °C; whereas, the mixed culture M3 maintained the yeast ratios at 22 °C, but not at 27 °C. In contrast, mixed cultures M1 and M4 did not maintain their inoculated yeast ratios at either 22 °C or 27 °C.

Interestingly, strains A1, A2 and A3 were present in all mixtures fermented at 22 °C and 27 °C, despite the tendency of strain A3 to exceed its inoculated ratio at the midpoint of the fermentation (Figure 2c,d). If a mixed-Burgundian yeast product were to be commercialized, the fermentation kinetics of the three isolates A1, A2 and A3 would need to be more thoroughly examined, both individually and in combination, in order to optimize performance.

### 2.2. Enological Characterization of Wine Yeast Strains

Analyses of variance (ANOVA) demonstrated that six of the eight enological characteristics varied significantly among the yeasts (Table 1). Two characteristics, ethanol and sugar/ethanol ratio, with values of 13.39%–13.77% v/v and 0.464–0.482, respectively, were not different among the yeasts.

Glycerol production by the individual- and mixed-Burgundian isolates fell within the range (8–10.5 g·L^−1^) associated with industrial yeasts (Table 1); this demonstrated their enological equivalence and suitability for winemaking. Wines from A1 at 22 °C, had a higher glycerol concentration than the other individual- and mixed-Burgundian wines (Table 1). Thus, this yeast would be looked upon favorably by winemakers, for glycerol increases osmotolerance and shunts carbon away from ethanol production [22].

In contrast, the individual- and mixed-Burgundian isolates produced amongst the lowest acetic acid concentrations, with the commercial strain BGY producing the highest concentrations at both 22 °C and 27 °C (Table 1). However all concentrations were below the red wine aroma detection threshold (0.6–0.9 g·L^−1^) [23] and beneath the US legal limit of 1.2 g·L^−1^. Therefore, all isolates and their mixtures were acceptable for commercial winemaking.

Ethanol tolerance of all yeast strains was within a single percentage point of one another at 22 °C (18.15%–18.90% v/v) and 27 °C (17.35%–17.75% v/v) (Table 1). Two industrial strains (AMH, BGY) were markedly less tolerant than the other yeast strains, producing 17.68 and 18.21% (v/v) ethanol at 22 °C, and 15.42 and 16.33% (v/v) ethanol at 27 °C, respectively. At both temperatures, the individual- and mixed-Burgundian isolates had intermediate ethanol tolerances, reflecting that they were enologically similar to the industrial strains.

Final optical density (growth phenotype) of the yeasts revealed that the Burgundian isolate (A1), and its mixtures (M1–M4), showed aberrant overall growth pattern (Table 1), with a complete scattering of OD measurements upon reaching the stationary phase due to flocculation. Flocculation of yeast cells is considered desirable in winemaking [8] for it allows for easier exclusion of yeast sediment during racking [24]. This characteristic could be particularly important for the production of premium unfiltered red wines. In contrast, BGY had a visibly longer lag phase at 27 °C; this was somewhat undesirable for it would allow other yeasts to establish and potentially dominate the fermentation. Interestingly, Enoferm Assmanshausen (AMH) reached only 75% of the final cell density of the other strains (Table 1), which were similar. Based on growth phenotype, the Burgundian isolates and their mixtures were considered enologically equivalent, as long as the late flocculation of strain A1 could be manageable in the cellar.

Foam production of the Burgundian isolates (22 °C, 15.7–16.3 mm; 27 °C, 20.3–32.3 mm) fell within the range produced by the industrial strains (22 °C, 9.7–34.0 mm; 27 °C, 7.3–28.0 mm) (Table 1), with the individual-Burgundian isolates (A1, A2) producing foam at the higher end of this range. While they did not differ significantly from two industrial strains (BGY, RC212), further investigations would be necessary to assess their foam production in other musts and to establish the necessary tank requirements [24].

**Table 1:** Mean ^a,b^ enological characteristics (glycerol, acetic acid, ethanol tolerance, final optical density, foam height, sulfur dioxide) for Pinot noir fermented with industrial strains and individual- and mixed-Burgundian *S. cerevisiae* isolates, at 22 °C and 27 °C. For each determination, strain and temperature effects are shown with subscripts and *p* values, respectively.

**Yeast**		**Glycerol (g·L^−1^)**		***p*^c^**	**Acetic Acid (g·L^−1^)**		***p***	**Ethanol Tolerance ^d^**		***p***
**Strain or Isolate**		**22 °C**	**27 °C**		**22 °C**	**27 °C**		**22 °C**	**27 °C**	
**Industrial**	**AMH**	8.79 d	10.02 d	***	0.124 b	0.187 cde	***	17.68 a	15.42 a	***
	**AWRI796**	9.34 e	10.64 e	*	0.132 c	0.179 bcd	*	18.30 bc	17.66 ef	**
	**BGY**	8.80 d	9.65 bcd	**	0.337 j	0.411 h	**	18.21 b	16.32 b	***
	**RA17**	8.43 bc	9.31 ab	*	0.224 h	0.292 g	***	18.56 cd	17.59 e	***
	**RC212**	7.94 a	9.12 a	**	0.246 i	0.250 f	ns	18.90 e	17.58 e	***
**Individual Burgundian**	**A1**	8.49 bcd	9.25 a	**	0.119 a	0.142 a	**	18.20 b	17.56 de	***
	**A2**	9.19 e	9.63 bc	**	0.209 g	0.240 f	*	18.73 de	17.35 c	***
	**A3**	8.44 bc	9.41 ab	***	0.135 c	0.167 b	*	18.15 b	17.42 cd	***
**Mixed Burgundian**	**M1 (1:1:1)**	8.37 b	9.33 ab	***	0.147 d	0.187 cde	**	18.55 cd	17.75 f	***
	**M2 (1:2:3)**	8.46 bc	9.85 cd	**	0.160 e	0.194 de	ns	18.40 bc	17.64 ef	***
	**M3 (3:2:1)**	8.48 bcd	9.37 ab	**	0.149 d	0.171 bc	**	18.57 cd	17.72 ef	***
	**M4 (1:3:2)**	8.72 cd	9.65 bcd	**	0.177 f	0.200 e	**	18.50 cd	17.75 f	*
	**Range**	7.94–9.34	9.12–10.64		0.119–0.337	0.142–0.411		17.68–18.73	15.42–17.75	
**Yeast**		**Final Optical Density ^e^**		***p***	**Foam Height ^f^ (mm)**		***p***	**Sulfur Dioxide ^f^ (mg·L^−1^)**		***p***
**Strain or Isolate**		**22 °C**	**27 °C**		**22 °C**	**27 °C**		**22 °C**	**27 °C**	
**Industrial**	**AMH**	1.494 a	1.667 a	**	9.7 a	7.3 a	ns	15.0 ab	12.5 ab	ns
	**AWRI796**	1.938 d	1.914 bc	ns	10.0 a	18.0 b	*	9.2 a	12.1 ab	ns
	**BGY**	1.658 b	1.841 b	ns	34.0 b	28.0 de	ns	9.9 a	6.4 a	ns
	**RA17**	1.931 d	1.992 c	***	13.3 a	20.3 bc	**	24.8 c	50.3 f	**
	**RC212**	1.940 d	1.989 c	*	16.3 a	24.0 cd	ns	36.5 d	28.4 cd	ns
**Individual Burgundian**	**A1**	n/a	n/a		16.0 a	28.3 de	ns	26.1 c	40.9 ef	***
	**A2**	1.819 c	1.831 b	ns	15.7 a	32.3 e	***	32.6 d	30.4 de	ns
	**A3**	1.861 cd	1.979 c	**	16.3 a	20.3 bc	ns	20.6 bc	18.2 bc	ns
**Mixed Burgundian**	**M1-M4**	n/a	n/a		n/a	n/a		n/a	n/a	
	**Range**	1.494–1.938	1.667–1.992		9.7–34.0	7.3–32.3		9.2–36.5	6.4–50.3	

^a^ The mean values of the biological replicates of each yeast strain were shown (*n* = 3). ^b^ Yeast strain means not sharing the same subscript are significantly (*p* ≤ 0.05) different at each temperature. ^c^ ns, *, **, *** indicates non-significant and significant at *p* ≤ 0.05, 0.01 and 0.001, respectively, for temperature effects. ^d^ Ethanol tolerance (% v/v) was defined as the ethanol produced from a high sugar must. ^e^ The final optical density (A_600_) of A1 was not available (n/a) due to complete light scattering due to flocculation. ^f^ Foam height and sulfur dioxide concentrations were assessed using additional fermentations.

Although sulfur dioxide production is generally not considered a selection criterion for wine yeasts, over abundance could inhibit the growth or cause stuck malolactic fermentations. Yeast strains in this research produced SO_2_ concentrations in the range of 10–50 mg·L^−1^ (Table 1). Although this was slightly higher than the usual (10–30 mg·L^−1^), it was believed attributed to the need to use a nutrient-poor synthetic grape must. Interestingly, the individual Burgundian isolates produced more SO_2_ than AMH, AWRI796, and BGY, but similar concentrations to Lalvin RA17 (RA17) and Lalvin Bourgorouge RC212 (RC212) (Table 1). The sulfur dioxide produced by the Burgundian isolates would not be expected to adversely impact malolactic fermentations, for most *O. oeni* strains can tolerate 15 mg·L^−1^ free- and 60–100 mg·L^−1^ total SO_2_, To verify this, assays were conducted to quantify malic acid consumption and lactic acid production for the Pinot noir fermentations (data not shown). Wines produced by the Burgundian strains completed malolactic fermentation as expected and had similar malic and lactic acid concentrations as the majority of the commercial strains. Therefore the Burgundian strains were considered enologically equivalent.

### 2.3. Analysis of Volatile Compounds

GC-MS of the headspace volatile compounds of Pinot noir wines revealed 25 quantifiable compounds. Analysis of variance (ANOVA) showed significant differences among the yeasts for nine higher alcohols (Table 2), seven ethyl esters (Table 3) and five acetate esters, two aldehydes, one acid and one acetal (Table 4) at 22 °C and 27 °C, except for 1-hexanol at 27 °C (Table 3), phenylethanol at 27 °C (Table 2) and hexyl acetate at 22 °C (Table 4). While differences for a particular yeast were really apparent, differences among the classes of yeast (industrial, individual- and mixed-Burgundian) were not. As such, it was desirable to use a multivariate statistical tool, principal component analysis (PCA), in order to extract the pattern of volatiles among the yeasts and between the temperatures. PCA allowed all 25 volatiles (higher alcohols, ethyl esters, acetate esters, aldehydes, acid, acetal) to be considered together.

**Figure 3:**
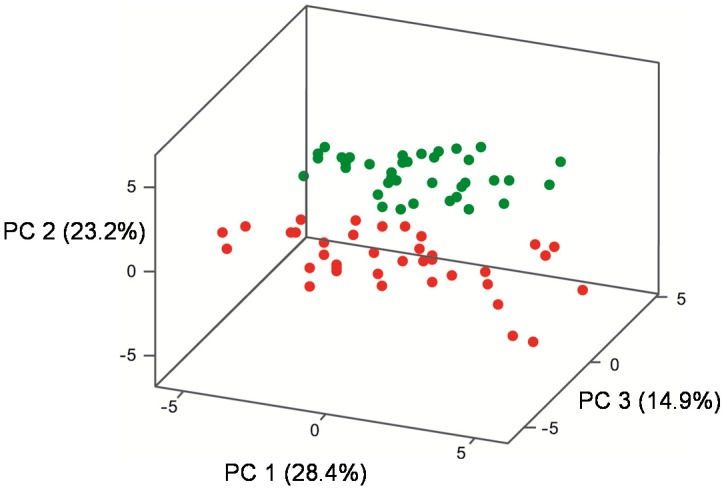
Principal component analysis (PC 1, PC 2, PC 3) of the mean concentration of 25 volatile compounds (nine higher alcohols, seven ethyl esters, five acetate esters, two aldehydes, one acid, one acetal) in Pinot noir, fermented using five industrial strains, three individual- and four mixed-Burgundian *S. cerevisiae* isolates in triplicate, at 22 °C (green) and 27 °C (red).

Principal components (PC) 1, 2 and 3 explained 28.4%, 23.2%, and 14.9% of the variation in the data set, respectively (Figure 3), with the vector loadings for the volatile compounds provided in Table 5. Volatile compounds with loadings greater than 1.2 were considered ‘heavily loaded’ and accounted for the majority of the variation among the yeasts.

**Figure 4:**
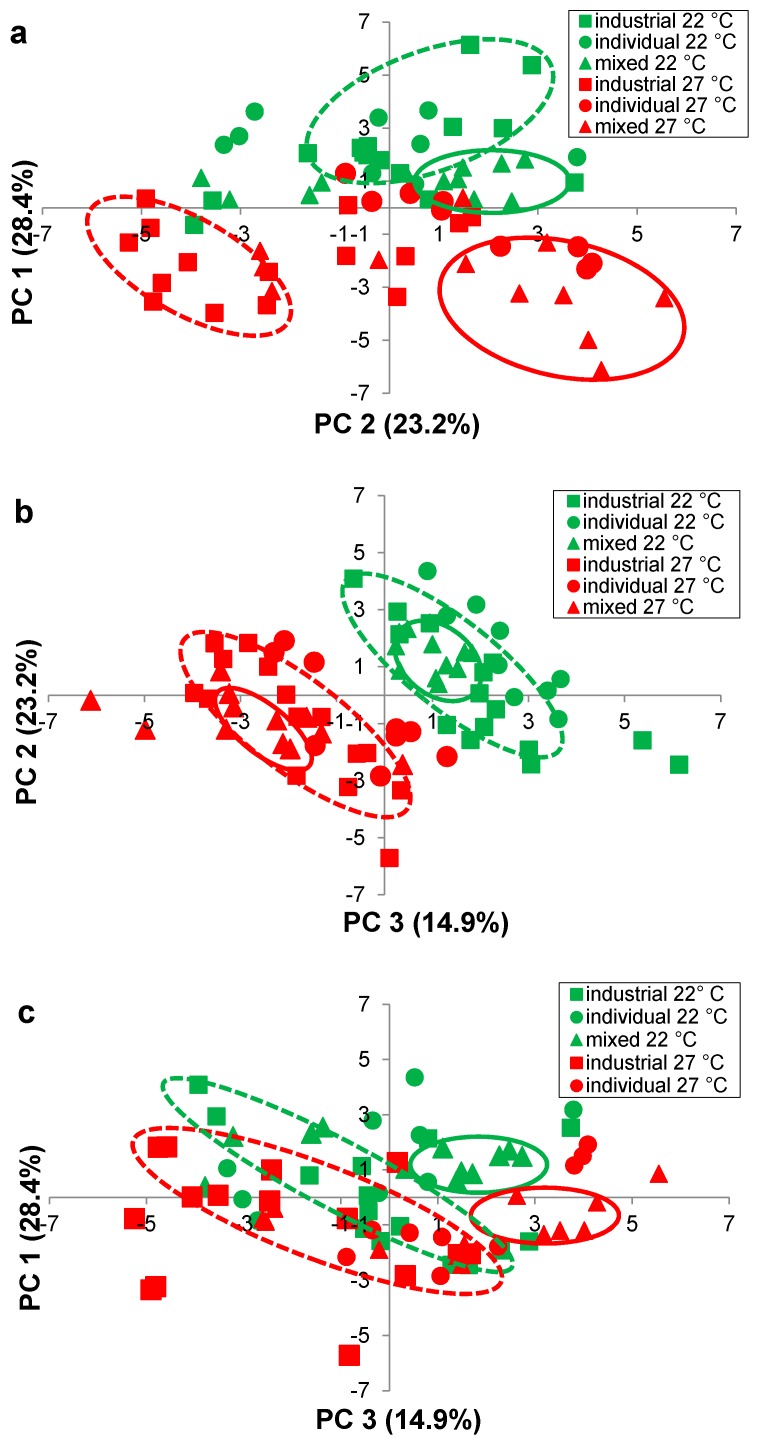
Principal component analysis (PC 1, PC 2, PC 3) of the mean concentration of 25 volatile compounds in Pinot noir, fermented using five industrial yeast strains (squares), three individual-Burgundian (circles) and four mixed-Burgundian (triangles) *S. cerevisiae* isolates in triplicate, at 22 °C (green) and 27 °C (red) (**a**) plot of PC 1 *versus* PC 2 (**b**) plot of PC 1 *versus* PC 2 and (**c**) plot of PC 1 *versus* PC 3. Ellipses drawn around wine fermented with industrial strains (dashed lines) and mixed-Burgundian isolates (solid lines), at 22 °C (green) and 27 °C (red), are to aid in discussion only.

Wines were clearly grouped according to their fermentation temperature (22 °C, 27 °C) (Figure 3), but yeast groups were more difficult to discern in a three-dimensional plot. Therefore, two-dimensional plots (Figure 4a–c) were prepared showing PC 1 vs PC 2 (Figure 4a), PC 2 *versus* PC 3 (Figure 4b) and PC 1 *versus* PC 3 (Figure 4c).

In Figure 4a, PC 1 was most heavily loaded in the positive direction with five ethyl esters (ethyl octanoate, ethyl hexanoate, ethyl butanoate, ethyl laurate, ethyl decanoate) and three acetate esters (ethyl acetate, hexyl acetate, isobutyl acetate, isoamyl acetate), as denoted by the high positive PC 1 values (Table 5). PC 1 was most heavily loaded in the negative direction with three higher alcohols (2-methyl-1-butanol, phenylethanol, 1-hexanol), as denoted by the negative PC 2 values (Table 5). Wine located on the lower left hand side of the plot (Figure 4a), such as the industrial wines fermented at 27 °C, had higher concentrations of these higher alcohols, while the remaining wines located on the right hand side of the plot (Figure 4a), had higher concentrations of the aforementioned ethyl and acetate esters.

**Table 2:** Higher alcohols (mg·L^−1^) ^a,b^ in Pinot noir fermented with industrial strains and individual- and mixed-Burgundian *S. cerevisiae* isolates at 22 °C and 27 °C. For each determination, strain and temperature effects are shown with subscripts and *p* values, respectively.

**Yeast**		**1,3-butanediol**		***p*^c^**	**2,3-butanediol**		***p***	**2-methyl-1-butanol**		***p***
**Strain or Isolate**		**22 °C**	**27 °C**		**22 °C**	**27 °C**		**22 °C**	**27 °C**	
**Industrial**	**AMH**	3.150 bcde	7.216 e	*	0.929 bcde	2.001 e	*	1.693 a	2.024 a	ns
	**AWRI796**	2.651 abcde	5.585 cd	**	0.854 abcd	1.497 cd	*	2.089 bcd	2.416 bcd	ns
	**BGY**	2.773 abcde	4.336 ab	**	0.913 bcde	1.213 abc	**	2.433 ef	2.481 cd	ns
	**RA17**	3.359 de	5.219 bcd	*	1.000 cde	1.405 bcd	*	1.951 ab	2.392 bcd	ns
	**RC212**	2.392 abcd	3.886 a	***	0.773 abc	1.050 a	**	2.053 bc	2.674 d	**
**Individual Burgundian**	**A1**	2.112 a	4.226 ab	***	0.669 a	1.166 ab	***	2.357 def	2.282 abc	ns
	**A2**	2.317 abc	4.663 abc	**	0.790 abc	1.322 abcd	**	2.315 cdef	2.140 ab	ns
	**A3**	2.239 ab	3.818 a	***	0.748 ab	1.057 a	***	2.090 bcd	2.074 a	ns
**Mixed Burgundian**	**M1 (1:1:1)**	3.610 e	5.893 d	*	1.110 e	1.524 d	ns	2.306 cdef	2.309 abc	ns
	**M2 (1:2:3)**	3.554 e	5.801 d	*	1.087 de	1.549 d	*	2.265 cde	2.484 cd	*
	**M3 (3:2:1)**	3.234 cde	5.734 cd	**	0.977 bcde	1.510 cd	**	2.330 cdef	2.278 abc	ns
	**M4 (1:3:2)**	3.575 e	5.187 bcd	*	1.075 de	1.343 abcd	ns	2.580 f	2.299 abc	ns
	**Range**	2.112–3.651	3.818–7.216		0.669–1.110	1.050–2.001		1.693–2.580	2.024–2.674	
**Yeast**		**3-methyl-1-butanol**		***p***	**Butanol**		***p***	**1-hexanol**		***p***
**Strain or Isolate**		**22 °C**	**27 °C**		**22 °C**	**27 °C**		**22 °C**	**27 °C**	
**Industrial**	**AMH**	8.229 a	9.309 a	ns	0.185 cd	0.270 cd	***	2.206 ab	2.520	ns
	**AWRI796**	9.840 bcd	10.815 bcd	ns	0.169 bc	0.317 e	***	2.413 abc	2.354	ns
	**BGY**	10.949 de	11.003 cd	ns	0.169 bc	0.212 a	*	2.649 cd	2.448	ns
	**RA17**	9.486 b	11.004 cd	ns	0.157 ab	0.225 ab	**	2.164 a	2.275	ns
	**RC212**	9.605 bc	11.843 d	*	0.263 f	0.517 f	***	2.214 ab	2.600	ns
**Individual Burgundian**	**A1**	11.518 e	10.888 bcd	ns	0.147 a	0.227 ab	***	2.499 bc	2.096	*
	**A2**	10.736 cde	9.745 ab	*	0.208 e	0.280 cd	*	2.497 abc	2.110	*
	**A3**	9.921 bcd	9.360 a	ns	0.189 cde	0.290 de	***	2.351 abc	2.005	*
**Mixed Burgundian**	**M1 (1:1:1)**	10.693 bcde	10.648 bcd	ns	0.184 cd	0.271 cd	***	2.54 bcd	2.171	ns
	**M2 (1:2:3)**	10.492 bcde	11.197 cd	*	0.194 de	0.254 bc	*	2.487 abc	2.393	ns
	**M3 (3:2:1)**	10.828 cde	10.541 abc	ns	0.171 bc	0.272 cd	***	2.533 bcd	2.151	ns
	**M4 (1:3:2)**	11.712 e	10.209 abc	ns	0.203 de	0.261 cd	*	2.841 d	2.187	*
	**Range**	8.229–11.712	9.309–11.843		0.169–0.263	0.227–0.317		2.164–2.841	2.005–2.600	
**Yeast**		**Isobutanol**		***p***	**Phenylethanol**		***p***	**Propanol**		***p***
**Strain or Isolate**		**22 °C**	**27 °C**		**22 °C**	**27 °C**		**22 °C**	**27 °C**	
**Industrial**	**AMH**	31.380 a	28.825 a	ns	0.472 a	0.827	**	12.845 f	11.804 e	ns
	**AWRI796**	50.592 bc	53.129 b	ns	0.709 cd	0.783	ns	11.198 de	12.111 e	ns
	**BGY**	71.731 d	72.157 d	ns	0.680 bcd	0.829	ns	8.654 ab	7.456 a	ns
	**RA17**	53.548 bc	66.743 cd	ns	0.565 ab	0.745	ns	8.179 a	7.837 ab	ns
	**RC212**	72.158 d	110.846 f	**	0.570 ab	0.732	*	8.221 a	9.086 cd	ns
**Individual Burgundian**	**A1**	62.119 cd	87.520 e	**	0.652 bc	0.672	ns	13.487 f	14.164 f	ns
	**A2**	44.142 b	55.413 b	ns	0.623 bc	0.618	ns	9.555 bc	9.217 cd	ns
	**A3**	43.416 ab	51.732 b	*	0.632 bc	0.641	ns	8.815 ab	7.767 ab	*
**Mixed Burgundian**	**M1 (1:1:1)**	58.954 c	56.836 bc	ns	0.645 bc	0.682	ns	10.984 de	9.908 d	ns
	**M2 (1:2:3)**	56.976 c	56.554 bc	ns	0.636 bc	0.746	ns	9.989 c	8.589 bc	ns
	**M3 (3:2:1)**	60.020 cd	58.112 bc	ns	0.713 cd	0.661	ns	11.775 e	11.122 e	ns
	**M4 (1:3:2)**	53.698 bc	51.769 b	ns	0.804 d	0.673	ns	10.258 cd	8.380 abc	**
	**Range**	31.380–72.158	28.825–87.520		0.472–0.804	0.661–0.829		8.179–12.845	7.456–14.164	

^a^ the mean values of the biological replicates of each yeast strain were shown (*n* = 3). ^b^ yeast strain means not sharing the same subscript are significantly (*p* ≤ 0.05) different at each temperature. ^c^ ns, *, **, *** indicates non-significant and significant at *p* ≤ 0.05, 0.01 and 0.001, respectively, for temperature effects.

**Table 3:** Ethyl esters (mg·L^−1^) ^a,b^ in Pinot noir fermented with industrial strains and individual- and mixed-Burgundian *S. cerevisiae* isolates, at 22 °C and 27 °C. For each determination, strain and temperature effects are shown with subscripts and *p* values, respectively.

**Yeast**		**Ethyl butanoate**		***p*^c^**	**Ethyl decanoate**		***p***	**Ethyl hexanoate**		***p***
**Strain or Isolate**		**22 °C**	**27 °C**		**22 °C**	**27 °C**		**22 °C**	**27 °C**	
**Industrial**	**AMH**	2.689 ab	1.707 a	***	0.031 d	0.020 ab	**	0.060 d	0.040 ab	***
	**AWRI796**	3.232 cde	2.327 bc	ns	0.025 cd	0.024 abc	ns	0.059 cd	0.043 bcd	*
	**BGY**	2.368 a	2.193 abc	ns	0.021 bc	0.017 a	ns	0.046 ab	0.036 a	*
	**RA17**	2.862 bcd	2.180 abc	***	0.022 bc	0.024 abc	ns	0.051 abc	0.041 abc	**
	**RC212**	3.181 cde	2.116 ^a^bc	*	0.024 c	0.023 abc	ns	0.052 abc	0.040 abc	ns
**Individual Burgundian**	**A1**	3.262 de	3.131 e	ns	0.025 cd	0.034 e	*	0.054 cd	0.050 d	ns
	**A2**	2.286 a	2.107 ab	ns	0.015 ab	0.031 cde	***	0.046 ab	0.046 cd	ns
	**A3**	3.387 e	2.893 de	*	0.023 c	0.026 bcde	ns	0.053 bcd	0.041 abc	**
**Mixed Burgundian**	**M1 (1:1:1)**	3.275 de	3.080 e	ns	0.024 c	0.030 cde	ns	0.054 cd	0.048 d	ns
	**M2 (1:2:3)**	3.316 e	2.626 cde	ns	0.024 c	0.024 abc	ns	0.053 bcd	0.042 abc	ns
	**M3 (3:2:1)**	3.252 de	3.117 e	ns	0.025 cd	0.033 de	ns	0.054 cd	0.050 d	ns
	**M4 (1:3:2)**	2.826 bc	2.494 bcd	ns	0.013 a	0.025 bcd	ns	0.045 a	0.041 abc	ns
	**Range**	2.368–3.316	1.707–3.131		0.013–0.031	0.017–0.034		0.045–0.060	0.036–0.050	
**Yeast**		**Ethyl lactate**		***p***	**Ethyl laurate**		***p***	**Ethyl octanoate**		***p***
**Strain or Isolate**		**22 °C**	**27 °C**		**22 °C**	**27 °C**		**22 °C**	**27 °C**	
**Industrial**	**AMH**	0.548 a	0.682 b	*	0.006 def	0.004 a	**	0.068 d	0.035 a	***
	**AWRI796**	0.847 ab	0.882 cd	ns	0.007 ef	0.008b cd	ns	0.053 bc	0.040 ab	ns
	**BGY**	1.773 c	0.671 b	ns	0.004 bcd	0.004 a	ns	0.046 abc	0.033 a	ns
	**RA17**	0.631 ab	0.726 b	ns	0.004 abc	0.005 ab	ns	0.047 abc	0.040 ab	ns
	**RC212**	0.753 ab	0.939 d	**	0.005 cd	0.006 ab	ns	0.046 abc	0.035 a	ns
**Individual Burgundian**	**A1**	0.973 b	0.936 d	ns	0.007 f	0.009 cd	**	0.057 cd	0.052 cd	ns
	**A2**	0.749 ab	0.671 b	ns	0.002 ab	0.007 bc	**	0.043 ab	0.052 cd	***
	**A3**	0.724 ab	0.719 b	ns	0.004 abc	0.006 ab	*	0.055 bc	0.040 ab	*
**Mixed Burgundian**	**M1 (1:1:1)**	0.817 ab	not quantifiable	***	0.005 cd	0.009 d	*	0.057 cd	0.048 bcd	ns
	**M2 (1:2:3)**	0.780 ab	0.799 bc	ns	0.004 cd	0.007 bcd	ns	0.056 cd	0.040 ab	ns
	**M3 (3:2:1)**	0.857 ab	0.766 bc	ns	0.005 cde	0.009 d	*	0.057 cd	0.055 d	ns
	**M4 (1:3:2)**	0.902 ab	0.698 b	ns	0.002 a	0.006 b	*	0.036 a	0.042 abc	ns
	**Range**	0.548–1.773	0.671–0.939		0.002–0.007	0.004–0.009		0.036–0.068	0.035–0.055	
**Yeast**		**Ethyl palmitate**		***p***						
**Strain or Isolate**		**22 °C**	**27 °C**							
**Industrial**	**AMH**	0.045 a	0.054 a	ns						
	**AWRI796**	0.118 ef	0.133 d	ns						
	**BGY**	0.072 abc	0.083 b	ns						
	**RA17**	0.068 abc	0.101 bc	*						
	**RC212**	0.103 def	0.143 d	ns						
**Individual Burgundian**	**A1**	0.123 f	0.145 d	ns						
	**A2**	0.056 ab	0.101 bc	*						
	**A3**	0.090 cde	0.101 bc	ns						
**Mixed Burgundian**	**M1 (1:1:1)**	0.105 ef	0.139 d	ns						
	**M2 (1:2:3)**	0.108 ef	0.120 cd	ns						
	**M3 (3:2:1)**	0.114 ef	0.127 cd	ns						
	**M4 (1:3:2)**	0.077 bcd	0.090 b	ns						
	**Range**	0.045–0.123	0.054–0.133							

^a^ the mean values of the biological replicates of each yeast strain were shown (*n* = 3). ^b^ yeast strain means not sharing the same subscript are significantly (*p* ≤ 0.05) different at each temperature. ^c^ ns, *, **, *** indicates non-significant and significant at *p* ≤ 0.05, 0.01 and 0.001, respectively, for the temperature effects.

**Table 4:** Acetate esters, aldehydes, acid and acetal (mg·L^−1^) ^a,b^ in Pinot noir fermented by industrial strains and individual- and mixed-Burgundian *S. cerevisiae* isolates, at 22 °C and 27 °C. For each determination, strain and temperature effects are shown with subscripts and *p* values, respectively. For each determination, strain and temperature effects are shown with subscripts and *p* values, respectively.

**Yeast**		**Ethyl acetate**		***p*^c^**	**Hexyl acetate**		***p***	**Isoamyl acetate**		***p***
**Strain or Isolate**		**22 °C**	**27 °C**		**22 °C**	**27 °C**		**22 °C**	**27 °C**	
**Industrial**	**AMH**	10.039 bc	8.968 ab	ns	0.029	0.017 a	ns	0.203 abcd	0.148 a	ns
	**AWRI796**	9.889 bc	10.191 bcd	**	0.022	0.020 ab	ns	0.188 abc	0.213 bcd	ns
	**BGY**	10.083 bc	9.636 abc	ns	0.016	0.018 a	ns	0.171 a	0.184 abc	ns
	**RA17**	9.944 bc	10.111 bcd	*	0.027	0.027 abcd	ns	0.228 bcd	0.255 def	ns
	**RC212**	8.824 a	8.615 a	ns	0.022	0.016 a	ns	0.190 abc	0.178 ab	ns
**Individual Burgundian**	**A1**	10.467 c	11.271 de	ns	0.024	0.036 d	ns	0.235 cd	0.320 g	*
	**A2**	9.335 ab	10.342 bcd	*	0.015	0.033 cd	***	0.167 a	0.267 defg	***
	**A3**	10.077 bc	9.342 ab	ns	0.025	0.025 abcd	ns	0.211 abcd	0.222 bcd	ns
**Mixed Burgundian**	**M1 (1:1:1)**	10.613 c	11.471 de	ns	0.027	0.030 bcd	ns	0.234 cd	0.291 efg	ns
	**M2 (1:2:3)**	10.850 c	10.994 cde	*	0.027	0.020 ab	ns	0.237 d	0.225 bcd	ns
	**M3 (3:2:1)**	10.599 c	11.807 e	ns	0.027	0.032 cd	ns	0.241 d	0.305 fg	ns
	**M4 (1:3:2)**	10.360 c	9.751 abc	ns	0.016	0.024 abc	ns	0.182 ab	0.241 cde	ns
	**Range**	8.824–10.850	8.615–11.807		0.016–0.029	0.016–0.033		0.167–0.241	0.148–0.320	
**Yeast**		**Isobutyl acetate**		***p***	**Methyl acetate**		***p***	**Acetaldehyde**		***p***
**Strain or Isolate**		**22 °C**	**27 °C**		**22 °C**	**27 °C**		**22 °C**	**27 °C**	
**Industrial**	**AMH**	0.00091 a	0.00071 a	**	0.889 a	0.784 a	ns	1.026 cd	0.967 bcd	ns
	**AWRI796**	0.00105 ab	0.00137 b	ns	1.245 cd	1.041 bc	ns	0.504 a	0.614 a	*
	**BGY**	0.00148 de	0.00187 cd	ns	1.105 bc	1.077 bc	ns	0.670 a	0.690 ab	ns
	**RA17**	0.00125 bcd	0.00194 cde	ns	0.996 ab	0.932 ab	ns	1.339 e	1.357 ef	ns
	**RC212**	0.00158 e	0.00202 cde	**	1.304 d	1.126 cd	ns	0.871 bc	0.803 ab	ns
**Individual Burgundian**	**A1**	0.00155 e	0.00286 f	ns	1.311 d	1.175 cde	ns	0.839 b	0.947 bcd	ns
	**A2**	0.00096 a	0.00165 bc	**	0.946 a	1.017 bc	ns	0.979 bcd	1.156 cde	ns
	**A3**	0.00111 abc	0.00159 bc	ns	1.196 cd	1.006 bc	*	0.901 bcd	0.737 ab	ns
**Mixed Burgundian**	**M1 (1:1:1)**	0.00133 cde	0.00215 de	**	1.183 cd	1.252 de	ns	0.923 bcd	1.377 ef	***
	**M2 (1:2:3)**	0.00133 cde	0.00176 bcd	*	1.198 cd	1.335 e	ns	1.009 cd	1.538 f	ns
	**M3 (3:2:1)**	0.00140 de	0.00235 e	**	1.192 cd	1.333 e	ns	1.049 d	1.245 def	ns
	**M4 (1:3:2)**	0.00104 ab	0.00166 bc	ns	1.172 cd	1.065 bc	ns	0.937 bcd	0.939 bc	ns
	**Range**	0.0009–0.00155	0.00071–0.00235		0.889–1.311	0.784–1.335		0.504–1.339	0.737–1.538	
**Yeast**		**Benzaldehyde**		***p***	**Acetic acid**		***p***	**1,1-diethoxyacetal**		***p***
**Strain or Isolate**		**22 °C**	**27 °C**		**22 °C**	**27 °C**		**22 °C**	**27 °C**	
**Industrial**	**AMH**	0.029 ab	0.056 abcd	*	0.387 a	0.427 ab	ns	2.833 b	3.357 d	ns
	**AWRI796**	0.056 fg	0.061 bcdef	ns	0.394 a	0.375 a	**	2.049 a	2.364 b	ns
	**BGY**	0.045 de	0.056 abcd	*	0.662 b	0.658 cd	ns	1.885 a	2.808 c	ns
	**RA17**	0.063 g	0.068 f	ns	0.450 a	0.864 d	ns	4.677 e	4.827 f	ns
	**RC212**	0.050 ef	0.063 def	**	0.480 a	0.693 cd	ns	3.210 bcd	2.750 c	ns
**Individual Burgundian**	**A1**	0.034 bc	0.055 abc	***	0.332 a	0.586 bc	ns	3.303 bcd	3.569 d	ns
	**A2**	0.026 a	0.053 ab	**	0.439 a	0.347 a	ns	3.362 bcd	3.336 d	ns
	**A3**	0.025 a	0.052 a	***	0.356 a	0.847 d	ns	3.169 bc	2.373 b	ns
**Mixed Burgundian**	**M1 (1:1:1)**	0.039 cd	0.064 ef	***	0.445 a	0.726 cd	ns	3.294 bcd	not quantifiable	ns
	**M2 (1:2:3)**	0.037 cd	0.062 cde	**	0.415 a	0.533 abc	**	3.780 cd	not quantifiable	ns
	**M3 (3:2:1)**	0.043 de	0.057 abc	*	0.342 a	0.586 bc	**	3.909 d	4.163 e	ns
	**M4 (1:3:2)**	0.050 ef	0.059 abc	ns	0.383 a	0.629 bc	*	3.601 cd	3.498 d	ns
	**Range**	0.029–0.063	0.052–0.068		0.332–0.662	0.347–0.864		1.885–4.677	2.364–4.827	

^a^ the mean values of the biological replicates of each yeast strain were shown (*n* = 3). ^b^ yeast strain means not sharing the same subscript are significantly (*p* ≤ 0.05) different at each temperature. ^c^ ns, *, **, *** indicates non-significant and significant at *p* ≤ 0.05, 0.01 and 0.001, respectively, for temperature effects.

Two of the mixed-Burgundian isolate wines from M1 and M2 were positioned particularly low in the plane (Figure 4a), with extremely high positive PC 1 and negative PC 2 values (Table 5); these wines had very much higher concentration of 1,3-butanediol and 2,3-butandiol (Table 2) and benzaldehyde (Table 4). A separation of the wines by temperature can also be seen in Figure 4b. Wines fermented at 22 °C and 27 °C were grouped diagonally across the plot, primarily located in the under right and lower left, respectively. Those in the upper right (^+^PC 2, ^+^PC 3) (Table 5) had higher concentrations of ethyl hexanoate, ethyl butanoate and ethyl octanoate (Table 3), while those in the lower left (^−^PC 2, ^−^PC 3) (Table 5) had higher concentrations of 2,3-butanediol and 1,3-butanediol (Table 2), benzaldehyde (Table 4) and acetic acid (Table 4).

Wines on the right hand side were further differentiated along PC 2, in the upper (^+^PC 2) and lower (^−^PC 2) quadrants. Wines from the industrial stains and the individual- and mixed-Burgundian isolates fermented at 22 °C, were located primarily in quadrant 1 (Figure 4a) with positive PC 1 and PC 2 values (Table 5). These wines had higher concentration of three ethyl esters (ethyl octanoate, ethyl hexanoate, ethyl butanoate) (Table 3). Many of the wines fermented with the individual- and most of the mixed-Burgundian isolates at 27 °C were located in quadrant 4 (Figure 4a) with positive PC 1 and negative PC 2 values (Table 5). These wines had higher concentrations of three ethyl esters (ethyl laurate, ethyl decanoate, ethyl palmitate) (Table 3), five acetate esters (methyl acetate, ethyl acetate, hexyl acetate, isobutyl acetate, isoamyl acetate) (Table 4), as well as acetaldehyde (Table 4).

**Table 5:** Identification and principal component analysis factor loadings (PC 1, PC 2, PC 3) for 25 volatile compounds in the headspace of Pinot noir, fermented with industrial strains and individual- and mixed-Burgundian isolates.

Quadrant in Figure 4a	Volatile Compound	Volatile Class	Loading ^a^ PC 1 (x) 28.4%	Loading ^a^ PC 2 (y) 23.2%	Loading ^a^ PC 3 (z) 14.9%	Temperature that volatile predominates
1	Propanol	Higher alcohol	1.10	0.20	1.02	Inconclusive
1	Ethyl octanoate	Ethyl ester	**2.11**	**1.25**	0.72	22 °C
1	Ethyl hexanoate	Ethyl ester	**1.76**	**1.44**	**1.29**	22 °C
1	Ethyl butanoate	Ethyl ester	**1.86**	0.62	**1.74**	22 °C
1	1,1-Diethyoxyacetal	Acetal	0.17	0.82	0.10	22 °C
3	2,3-Butanediol	Higher alcohol	−0.03	**−1.98**	**−1.93**	27 °C
3	Isobutanol	Higher alcohol	−0.24	**−1.35**	**1.83**	27 °C
3	Butanol	Higher alcohol	−0.42	**−1.70**	−0.49	27 °C
3	3-Methyl-1-butanol	Higher alcohol	−0.88	**−1.69**	**2.38**	27 °C
3	2-Methyl-1-butanol	Higher alcohol	**−1.19**	**−1.88**	**1.96**	27 °C
3	Phenylethanol	Higher alcohol	**−1.23**	**−1.86**	0.70	27 °C
3	1-Hexanol	Higher alcohol	**−1.62**	−0.23	2.07	Inconclusive
3	Ethyl lactate	Ethyl ester	−0.80	−0.03	2.09	Inconclusive
3	Benzaldehyde	Aldehyde	−0.03	**−2.22**	−1.09	27 °C
3	Acetic acid	Acid	−0.14	**−1.88**	−1.08	27 °C
4	1,3-Butanediol	Higher alcohol	0.10	**−2.10**	**−1.97**	27 °C
4	Ethyl laurate	Ethyl ester	**1.93**	**−1.46**	0.00	27 °C
4	Ethyl decanoate	Ethyl ester	**2.35**	−0.43	−0.41	27 °C
4	Ethyl palmitate	Ethyl ester	1.00	**−1.87**	**1.48**	27 °C
4	Methyl acetate	Acetate ester	1.01	−0.96	**2.45**	27 °C
4	Ethyl acetate	Acetate ester	**1.95**	−0.96	0.73	27 °C
4	Hexyl acetate	Acetate ester	**2.45**	−0.02	−0.51	Inconclusive
4	Isobutyl acetate	Acetate ester	**1.36**	**−1.89**	0.42	27 °C
4	Isoamyl acetate	Acetate ester	**2.31**	−0.95	−0.07	27 °C
4	Acetaldehyde	Aldehyde	1.10	−0.90	−1.12	27 °C

^a^ coordinates in bold font, with absolute values greater than 1.2, were most heavily loaded.

The mixed-Burgundian isolates formed smaller tighter subsets within the larger groupings (Figure 4b), suggesting that they were more similar to one another than to the remaining wines. Such findings are consistent with Saberi *et al.* (2012) [5] who also reported that co-cultured wines were more similar to one another than to industrial strains. The similarity of mixed-Burgundian strain wines was also evident in Figure 4c. Wine fermented with the mixed-Burgundian at 22 °C (^+^PC 1, ^+^PC 3) (Table 5) had higher concentrations of ethyl esters (ethyl octanoate, ethyl hexanoate, ethyl butanoate, ethyl palmitate) (Table 3) and acetate esters (methyl acetate, ethyl acetate) (Table 4). In contrast the mixed-Burgundian wines at 27 °C (^+^PC 1, ^−^PC 3) (Table 5) had higher concentrations of acetate esters (hexyl acetate, isobutyl acetate and isoamyl acetate) (Table 4) and 1,3-butanediol (Table 2). Close examination of the volatiles produced from the individual-Burgundian isolates (Table 2, Table 3 and Table 4) revealed that the yeast with the lowest production at 22 °C was not necessarily the yeasts with the lowest production at 27 °C (higher alcohols, Table 2; ethyl esters, Table 3; acetate esters/aldedhydes/acid/acetal, Table 4). This suggested that the strain differences, while significant, may be subtle compared to the magnitude of the temperature differences. For example, the low concentration of 3-methyl-1-butanol in the co-cultured wine M2 at 22 °C (10.49 mg·L^−1^, Table 2) reflected the concentration associated with the low producer A3 (9.921 mg·L^−1^, Table 2). Similarly, the low concentrations of ethyl butanoate (2.826 mg·L^−1^, Table 3) and ethyl acetate (0.00104 mg·L^−1^, Table 4) in M4 at 22 °C were consistent with the concentrations produced by the dominant yeast A2 (ethyl butanoate, 2.286 mg·L^−1^, Table 3; ethyl acetate 0.00096 mg·L^−1^, Table 4). Such results were consistent with the inoculation and fermentation ratios (Figure 2) and are a testament to the fact that co-cultured yeasts can be used to significantly modify the headspace volatiles of a wine. Such trends were not as readily apparent at 27 °C, in part due to the fact that the inoculation ratios were not as well maintained at this temperature.

The volatile compositions of the wine from the mixed-Burgundian isolates were unattainable by any single industrial yeast strain. Although the compound 2,3-butanediol may be present at relatively high concentrations (Table 2) [25], its contribution to wine aroma is somewhat elusive given its high detection threshold (150 mg·L^−1^) [26]. Similarly, propanol’s contribution to wine aroma is unclear [27]. However, it shows an inverse correlation with hydrogen sulfide [28], suggesting that wines from mixed-Burgundian isolates may have a lower propensity to sulfur flaws.

While the higher alcohols (1-hexanol, 3-methyl-1-butanol, 2-methyl-1-butanol) can contribute to wine quality at low concentrations (~300 mg·L^−1^), they can detract from wine quality at high concentrations (~400 mg·L^−1^) [29]. In the context of this study, it is difficult to determine whether the increased in concentration of these higher alcohols for the mixed-Burgundian isolates represents a positive, negative, or negligible impact on the sensory properties of the wine. Nevertheless, the differences in higher alcohol production and the unique combination of higher alcohols in wines fermented with mixed-Burgundian yeasts could indicate the future potential of mixed strain yeast products.

The industrial strains and mixed-Burgundian isolates at 27 °C had a propensity to produce slightly more ethyl esters during fermentation (Table 3). Esters are particularly important to wine aroma for they can be perceived sensorially. Although Ferreira *et al.* [30] suggests that acetate and ethyl esters may only play a modulatory role in red wine aroma, their contribution would be expected to be dependent on the style of red wine, particularly if the Pinot noir was prepared without skin contact as in this research Howell *et al.* [17] identified that co-culturing wines produced volatile profiles that could not be replicated by fermenting each strain individually, or by blending the wines from single cultures. This suggests that co-cultured yeasts may be sharing metabolites [31] and creating unique volatile profiles that are more than the sum of their parts. King *et al.* [16] reported that consumers, who were familiar with the higher priced wines, preferred wines that had been co-cultured with two yeast strains. Such findings are consistent with Saberi *et al.* [5] and Grossman *et al.* [32] who report that co-cultured wines had intermediate concentrations of odor active compounds and were perceived as more complex, respectively. As such the isolates evaluated in this research offer winemakers an opportunity to produce wines with unique and/or more complex characters.

## 3. Experimental Section

### 3.1. Yeast and Bacterial Strains Employed

Three novel *S. cerevisiae* strains (A1, A2, A3) were isolated in 2007 from a vineyard in Burgundy France and preserved in 15% glycerol/yeast peptone dextrose (YPD) broth at −80 °C. These isolates were compared to five commercially available *S. cerevisiae* strains, which were recommended for Pinot noir fermentation. The industrial strains Enoferm Assmanshausen (AMH), Enoferm Burgundy (BGY), Lalvin RA17 (RA17) and Lalvin Bourgorouge RC212 (RC212) were purchased as active dry yeast from Lallemand Inc. (Rexdale, ON, Canada); whereas Australian Wine Research Institute 796 (AWRI796) was obtained as an agar slant from Mauri Yeast Australia (Sydney, Australia).Yeast for the killer phenotyping assay, *S. cerevisiae* wine strains EC 1118 and UCD 522 (Montrachet), were obtained from freezer stocks maintained by the van Vuuren laboratory. The malolactic *O. oeni* bacterial strain Lalvin 31 was purchased from Lallemand Inc. The individual-Burgundian isolates were prepared in four mixtures (M1, M2, M3, M4) consisting of the ratios of the isolates A1, A2 and A3 as follows: 1:1:1, 1:2:3, 3:2:1 and 1:3:2, respectively.

### 3.2. Media and Culture Conditions

All *S. cerevisiae* strains were maintained as freezer stocks at −80 °C in 15% glycerol/YPD and cultured in Difco YPD broth and agar (Becton, Dickinson and Co., Franklin Lakes, NJ, USA) according to standard procedures [33]. Lyophilized *O. oeni* was rehydrated in 50 mL of sterile distilled water for 15 min and used directly for the malolactic fermentation compatibility study.

Killer assay medium was formulated by buffering YPD agar with 50 mM dibasic phosphate and adjusting the pH to 4.2 with citric acid prior to autoclaving. Filter sterile (0.22 µm) methylene blue was added at a rate of 0.0015% w/v (adapted from van Vuuren and Wingfield [34]).

Free run Pinot noir and Chardonnay grape must (2008) were obtained from Calona Vineyards (Kelowna, BC, Canada). It had been crushed, pressed and treated with ~50 mg kg^−1^ sulfur dioxide, then frozen prior to shipment to the Wine Research Centre (Vancouver, BC, Canada). Pinot noir must was thawed just prior to inoculation; its composition was: 25.2 °Brix, 3.77 pH, 5.62 g·L^−1^ titratable acidity (TA) and 244 mg·L^−1^ yeast available nitrogen (YAN). Chardonnay juice was used for the growth kinetic and phenotyping assays, since a lightly colored juice was required for the spectrophotometric determinations. Chardonnay juice was sterilized using a 0.22 µm filter; its composition was: 27.0 °Brix, 3.46 pH, 5.76 g·L^−1^ TA and 121 mg·L^−1^ YAN.

### 3.3. Genetic Fingerprinting and Monitoring of Mixed Strains During Fermentation

*S. cerevisiae* strains were genetically fingerprinted with the PCR method and the primers δ12 and δ2 described in Schuller *et al.* [18]. *S. cerevisiae* strains were grown and genomic DNA was extracted [33]. A 50 µL reaction mixture was prepared, which contained 10 ng of DNA template, 1 U iProof DNA polymerase (BioRad, Mississauga, ON, Canada), 5× GC buffer, 0.5% v/v DMSO, 0.2 mM of each dNTP, and 25 pmol of each primer. After the initial denaturation at 98 °C for 3 minutes, the reaction mixture was cycled 30 times according to the following program: 98 °C for 10 s, 55 °C for 1 min, and 72 °C for 1 min, which was followed by a final elongation period at 72 °C for 10 min. The PCR products were separated by gel electrophoresis on a 1.5% agarose gel and visualized with SYBR Safe DNA gel stain (Invitrogen Inc., Burlington, ON, Canada).

Wine fermentations containing the Burgundian isolates (A1, A2, A3) in the mixtures M1 (1:1:1), M2 (1:2:3), M3 (3:2:1), and M4 (1:3:2) were monitored with genetic fingerprinting of the individual visualized through colony PCR. Cells were harvested by centrifugation (5000 × *g* for 5 min) at the midpoint (9% v/v ethanol) and end (13.5% v/v ethanol) of fermentation. Cells were resuspended and diluted in sterile MilliQ water before being grown up on YPD agar plates at 30 °C for 3 d. The genetic fingerprints of 45 colonies from each replicate (n = 3) at each time point (n = 2) and temperature (n = 2) were assessed via colony PCR by substituting a small amount of colony for the DNA template in the method described above and increasing the initial denaturation period to 10 minutes.

### 3.4. Killer Factor Phenotyping

The killer factor phenotype was assessed in the individual-Burgundian isolates (A1, A2, A3) against the killer positive control (K^+^) *S. cerevisiae* strain EC1118, and killer negative control (K^−^) *S. cerevisiae* strain UCD522. All strains were grown on YPD-agar plates for 72 h at 30 °C. Three colonies of sensitive strain AMH were picked and resuspended in sterile MilliQ water to give 5 × 10^8^ cells mL^−1^; 300 µL of this suspension was spread as a lawn on a plate containing killer assay medium and allowed to dry. Colonies from each of the other strains were swabbed and spread as a thick line on top of the killer lawn. The plate was then incubated at 18 °C for 5 d (adapted from van Vuuren and Wingfield [34].

### 3.5. Model Fermentations—Fermentation Characteristics

*S. cerevisiae* freezer stocks were used to inoculate 5 mL cultures of YPD, which were grown overnight in a rotary wheel to stationary phase at 30 °C. Flasks containing 50 mL of YPD were subsequently inoculated at a rate of 5 × 10^5^ cells mL^−1^ and grown aerobically in a shaker bath (180 rpm) for 24 h at 30 °C. Cells were harvested by centrifugation (5000× *g* for 5 min), washed with sterile MilliQ water, and resuspended in fermentation medium at a density of 5 × 10^8^ cells mL^−1^. Fermentations were inoculated in biological triplicate at a rate of 2 × 10^6^ cells mL^−1^. In the case of the mixed strain fermentations, yeast strains were not combined prior to inoculation of the fermentation medium. All fermentations were conducted in media bottles topped with rubber bungs and water-filled capped gas locks to ensure anaerobic conditions. Sampling occurred anaerobically by piercing the rubber bungs with 5-inch hypodermic needles (Air-Tite Products Co., Virginia Beach, VA, USA) attached to 3 mL syringes (Becton, Dickinson and Co., Franklin Lakes, NJ, USA) and extracting approximately 1 mL of sample.

The primary experimental fermentations were conducted in triplicate in 900 mL of Pinot noir must at 22 °C and 27 °C, respectively, and were used to assess fermentation kinetics, ethanol, glycerol, and acetic acid production, mixed strain population dynamics, and production of volatile compounds. Sampling occurred twice daily early in the early stage, daily in the intermediate stage, and every two days in the final stage of fermentation. Ethanol production, form formation and glycerol production were measured at each time while acetic acid and the volatile compounds were assessed at the end of fermentation. Fermentation samples were vortexed, centrifuged, and filter sterilized (0.22 µm) before compounds were analysed. After sugars were depleted, 100 mg·L^−1^ of potassium metabisulfite was added to the wine to protect against oxidation. Samples were stored at 4 °C until GC-MS analysis. The ethanol tolerance of the various *S. cerevisiae* strains was assessed by fermenting each strain in biological triplicate in a high sugar must. This must was created by supplementing the Pinot noir must to 33% sugar, using equi-molar amounts of glucose and fructose (Fisher Scientific, Ottawa, ON, Canada). Fermentations were sampled initially and after the fermentations were complete (21 d) and the concentration of ethanol determined and expressed in % v/v, as described above.

Sulfur dioxide production by yeasts was assayed following the alcoholic fermentation in biological triplicate in 200 mL of synthetic juice at 22 °C and 27 °C. Sulfur dioxide was quantified in technical triplicate according to manufacturer protocols using the “Total SO_2_” UV test kit from R-Biopharm (Darmstadt, Germany). Unfermented synthetic juice was also assayed to ensure that it was free from sulfite contamination.

The malolactic compatibility of the strains was assessed following the alcoholic fermentation in biological triplicate in 400 mL of Pinot noir must at 22 °C. Wines were inoculated with *O. oeni* strain MBR 31 and fermented at 20 °C. Samples were collected and analysed for malic and lactic acids at 3–4 d intervals for 18 d.

### 3.6. Growth Phenotype Assay

The growth phenotypes of the yeasts were assayed in a Bioscreen C Growth Chamber (Thermo-Labsystems) in filter sterilized (0.22 µm) Chardonnay juice (Calona Vineyards). The *S. cerevisiae* strains were grown to stationary phase in 5 mL cultures of YPD at 30 °C in a rotary wheel, harvested by centrifugation (5000× *g* for 5 min) and resuspended in Chardonnay juice. The juice was then inoculated at a rate of 5 × 10^5^ cells mL^−1^ and 150 µL aliquots were transferred in triplicate into a 100-well Bioscreen C optical plate (Thermo-Labsystems). The optical plate was placed in the growth chamber and grown for 96 h with continuous shaking at 22 °C and 27 °C. The OD (A_600nm_) was measured automatically each hour; data were compiled using the affiliated Biolink-DOS software.

### 3.7. Foam Production Assay

Foam production was assessed in yeasts at 22 °C and 27 °C using an assay modified from Regodón *et al.* [35]. Yeasts were cultured in preparation for fermentation and were inoculated into 18 × 150 mm test tubes containing 10 mL of Pinot noir juice. Foam height was monitored three times per day and the maximum height achieved was measured and recorded in millimeters.

### 3.8. Quantification of Compounds Using HPLC

Ethanol, glycerol and acetic acid were quantified according with an Agilent 1100 series HPLC (Agilent Technologies, Palo Alto, CA, USA) using a Supelcogel C-61OH 30 cm × 7.8 mm column (Sigma-Aldrich, Oakville, ON, Canada), an Agilent G1362A refractive index detector with positive polarity and Agilent LC-MS ChemStation revision A.09.03 software. The method consisted of a 23 min isocratic run of 0.1% phosphoric acid at 0.75 mL min^−1^ [36]. Peak monitoring was performed with an Agilent G1362A refractive index detector (Agilent, Santa Clara, CA, USA). Concentrations were determined for each of the three replicates from the standard curves. Glycerol and acetic acid concentrations were reported in g·L^−1^. Ethanol concentrations were reported in percentage (v/v), in order to be consistent with units utilized by the wine industry.

### 3.9. Identification and Quantification of Volatile Compounds Using GC-MS

GC-MS headspace analysis was used to analyze Pinot noir wine samples according to the method of Danzer *et al.* [37], without solid phase microextraction (SPME) as described in Husnik *et al.* [1]. An Agilent 6890N GC interfaced to a 5973N Mass Selective Detector along with a 60 m × 0.25 mm ID, 0.25 µm thickness DBWAX fused silica open tubular column (J&W Scientific, Folsom, CA, USA) were used to detect and quantify volatile compounds, which were analysed with Enhanced Chemstation software (MSD Chemstation Build 75, Agilent Technologies, Palo Alto, CA, USA) and identified with the Wiley7Nist05 library (Wiley and Sons, Hoboken, NJ, USA).

### 3.10. Statistical Analyses

One-factor ANOVA with replication were used to examine the ethanol, glycerol, acetic acid, sulfur dioxide and volatile effects among the yeast strains using MS Excel 2010 (Microsoft, Redmond, WA, USA). Differences among strains were differentiated using Fisher’s least significant difference (LSD) at *p* ≤ 0.05 and delineated using subscripts.

Principal component analysis (PCA) was used to examine the patterns of 25 volatile compounds associated with the wine products from the five industrial strains and three individual- and four mixed-Burgundian isolates, at both fermentation temperatures in triplicate using Minitab 16 (Minitab Inc., State College, PA, USA). Vector coordinates were scaled by a factor of five times to aid in visualization of the data. A principal component (PC) plot was prepared for the first three dimensions (3d-plot) in Minitab 16 (Minitab Inc., State College, PA, USA). Two dimensional figures (2-d plots) of PC 1 *versus* PC 2, PC 2 *versus* PC 3 and PC 1 *versus* PC 3 were prepared in MS Excel (Microsoft, Redmond, WA, USA). Ellipses were drawn on these plots around the industrial strains and mixed-Burgundian isolates as visual aids only.

## 4. Conclusions

This research demonstrated the three Burgundian *S. cerevisiae* isolates (A1, A2, A3) were genetically unique from five industrial strains (AMH, AWRI796, BGY, RA17, RC212), killer positive and compatible with malolactic bacteria. The individual- and mixed-cultures of these new isolates were demonstrated to be suitable for winemaking, since their enological characteristics fell within the range associated with the industrial strains.

ANOVA of the 25 volatile compounds (nine alcohols, seven ethyl esters, five acetate esters, two aldehydes, one acid, one acetal) revealed differed among the yeast strains. Principal component analysis revealed that the differences in the volatile profiles among the yeasts (industrial, individual- and mixed-Burgundian) were more subtle than those due to temperature. Mixed-Burgundian isolates at 22 °C and 27 °C produced lower concentrations of higher alcohols than industrial yeasts at 27 °C, creating wines with unique volatile profiles. In general, the mixed-Burgundian strain wines were more similar to one another than to the industrial strains, with higher concentrations of several ethyl ester and acetate esters. This research documented that co-culturing novel strains can produce wines with unique volatile profiles, without the risks of spontaneous fermentation. As such, a commercial multi-yeast starter culture could serve as a winemaking tool to increase wine complexity and improve wine differentiation in the marketplace. However, much research remains to be conducted to optimize performance of the co-cultured strains, understand the mechanisms of yeast-yeast interaction, evaluate the relative contribution of the strains to overall wine flavor, elucidate the volatile/non-volatile interactions, and understand the changes to volatiles during ingestion and consumption.
